# Wave instabilities in the presence of non vanishing background in nonlinear Schrödinger systems

**DOI:** 10.1038/srep07285

**Published:** 2014-12-03

**Authors:** S. Trillo, J. S. Totero Gongora, A. Fratalocchi

**Affiliations:** 1 Dipartimento di Ingegneria, Università di Ferrara, Via Saragat 1, 44122 Ferrara, Italy; 2 PRIMALIGHT, Faculty of Electrical Engineering; Applied Mathematics and Computational Science, King Abdullah University of Science and Technology (KAUST), Thuwal 23955-6900, Saudi Arabia

## Abstract

We investigate wave collapse ruled by the generalized nonlinear Schrödinger (NLS) equation in 1+1 dimensions, for localized excitations with non-zero background, establishing through virial identities a new criterion for blow-up. When collapse is arrested, a semiclassical approach allows us to show that the system can favor the formation of dispersive shock waves. The general findings are illustrated with a model of interest to both classical and quantum physics (cubic-quintic NLS equation), demonstrating a radically novel scenario of instability, where solitons identify a marginal condition between blow-up and occurrence of shock waves, triggered by arbitrarily small mass perturbations of different sign.

Wave collapse, i.e. the occurrence of blow up in a finite time or propagation distance^1^,^2^,^3^, is a general phenomenon that appears in many contexts including self-focusing in optics^4^,^5^ (for an extensive review and a historical perspective, see ref. 6), plasma waves^7^, Bose-Einstein condensates^8^,^9^, hydrodynamics^10^ and organic systems^11^. Indeed several dispersive models with critical or supercritical nonlinearity (e.g. generalized KdV or KP equations^12^, modified KP^13^, Zhakarov equation^14^, and discrete or nonlocal version of the nonlinear Schrödinger equation^15^,^16^) exhibit formation of a point singularity from a large class of initial data. The critical case is by far the most intriguing one due to the sensitivity of the collapse to perturbations. In this context the most studied case, namely the focusing cubic nonlinear Schrödinger (NLS) equation, which is critical in two transverse dimensions, is still a formidable ground to understand the dynamics of collapse^17^,^18^,^19^,^20^ in spite of long standing investigations^3^,^4^,^5^,^6^. However, many physical classical and quantum systems need to be described in terms of generalized NLS (gNLS) equation which accounts for higher-order nonlinearities. Indeed such higher-order terms arise from different physical mechanisms in nonlinear optics (saturation of optical susceptibilities of standard material^21^,^22^,^23^, local field effects^24^, tailoring of nonlinearities in a cooled gas^25^), dynamics of superuids^26^, or quantum condensed systems where they are related to higher-order atom-atom interactions^27^,^28^,^29^. In gNLS systems critical collapse can occur also in one transverse dimension when quintic (or higher-order) nonlinearity is effective^2^,^30^,^31^. Though the blow-up problem is generally addressed for bright (i.e., zero background) solutions, gNLS systems support translationally invariant solitary wave solutions with non-zero background, yet of bright type^32^,^33^,^34^,^35^,^36^ (complement of well known dark soliton or bubble type solutions^37^,^38^), for which the problem of blow-up have been overlooked. Our aim in this paper is to establish a novel criterion for collapse valid for solutions of this type, and study the dynamics across the threshold for blow-up. This allows us to reveal a new instability scenario where opposite behaviors, such as blowup or decay into a dispersive shock wave, can be controlled by means of an arbitrarily weak perturbation which controls the variation of the power (or mass) integral of a launched perturbed solitary wavepacket. We consider regimes for which the background itself is stable (otherwise modulation instability of the background becomes the main mechanism that affects the decay dynamics of the field).

The paper is organized as follows. In Section 2 we introduce the model and develop a sufficient criterion for collapse. In Section 3, we propose an approach to characterize non-collapsing solutions based on self-similar and semiclassical scaling arguments. The prediction of our analysis are confirmed by means of numerical simulations with reference to a specific model of physical interest in Section 4.

## The model and collapse criterion

We start from the following gNLS equation in dimensionless units 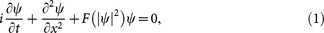
with a general nonlinearity *F*(*ρ*) (henceforth *ρ* = |*ψ*|^2^, which has physical meaning of density or intensity in optics) and study the instability scenario of localized fields *ψ* characterized by nonvanishing boundary conditions: 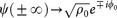. Equation (1) is conveniently normalized by setting *F*(*ρ*_0_) = 0 at the background value *ρ*_0_. Invariants of motion originate from the translational symmetries along *t*, *x* and Gauge transformations, resulting in the renormalized functionals associated with energy *H*, momentum *P*, and *L*^2^ norm *M* (henceforth referred to as ‘mass', though it can physically represent number of particles or power in optics)^39^

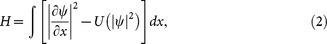

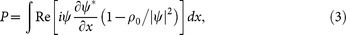

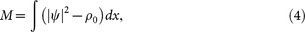
where 
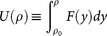
.

As demonstrated by Barashenkov^38^, traveling solitary solutions *ψ*(*x*, *t*) = *ϕ*(*x* − *vt*) of the gNLS equation (1) on a finite background *ρ*_0_ ≠ 0 are unstable when the slope of the curve *P* = *P*(*v*) becomes negative (*dP*/*dv* < 0). Under the validity of such assumption, we begin our theoretical analysis by finding a sufficient criterion for collapse based on virial identities (see the review papers in Refs. 1,2,3, 6. We address the most common regime where, in the absence of an initial derivative of the virial (second-order momentum), the collapse of a bright disturbance with vanishing background is known to be essentially ruled by the energy (Hamiltonian) invariant of motion. Conversely, for a finite background we show that blow-up is driven by a suitable combination of renormalized energy and mass, thus allowing the existence of non collapsing solutions even for negative energy. We then employ a combination of virial identities and arguments based on self-similarity to study the leading order scaling of the dynamics in both collapsing and non collapsing regimes. In the latter case, a suitable rescaling in a semiclassical form and a diagonalization of the resulting equations of motion in terms of Riemann invariants, allow us to predict a completely novel scenario where collapse is arrested and the instability leads to the decay into dispersive shock waves, i.e. expanding fast wavetrains that are naturally emitted, owing to dispersion, to regularize steep gradients developing through the nonlinearities^40^,^41^,^42^,^43^,^44^,^45^,^46^,^47^,^48^,^49^,^50^,^51^. We verified our theoretical finding on a model of physical interest, namely the defocusing-focusing Cubic-Quintic Nonlinear Schrödinger Equation (CQNLS). We demonstrate a specific class of localized pulses on a pedestal, represented by antidark solitons, marking a marginal condition crossover between collapse and shock-wave generation, controlled via (even infinitesimal) mass perturbations. These findings, other than the fundamental interest related to collapse, suggest a new avenue for arresting blow-up and generating dispersive shocks from unstable solutions with nonzero background.

We begin our theoretical analysis by writing the Hamilton-Jacobi equations of motion for the evolution of a suitable renormalized virial 
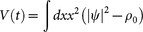
: 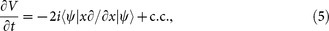

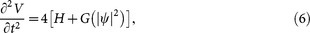
where we set 
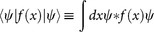
, and Eq. (6) is obtained by further deriving in time Eq. (5) and expressing such derivative in terms of the energy functional *H*, and the suitably defined functional 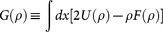, which depends on the nonlinear response of the system. In order to derive a sufficient criterion for collapse, we expand in series the functional *G*(*ρ*), obtaining 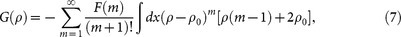
with 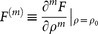. Several different scenario can arise according to the signs of the terms in the expansion (7). An interesting case manifests when a low order defocusing nonlinearity —which supports localized solutions on a pedestal— competes with high order contributions of different sign, i.e., when *F*^(1)^ < 0 and *F*^(*m*)^ > 0 for *m* > 2. The following inequality then holds 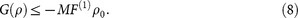
Then, combining Eq. (6) and (8), we find the following differential inequality 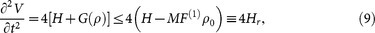
which gives an upper bound to the dynamics of the virial *V*(*t*) in terms of rescaled Hamiltonian *H_r_*, depending on the renormalized energy *H* and mass *M*. When *H_r_* < 0, or equivalently: 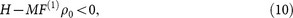
the virial *V*(*t*) ~ *Ct*^2^ (*C* negative constant) tends to zero regardless of any initial condition *V*(0) and *∂V*(0)/*∂t*. In order to understand the consequences of a vanishing virial on the dynamics of the field *ψ*, we employ a specific form of the Hölder inequality: 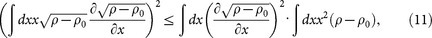
and through an integration by parts of Eq. (11), derive the following upper limit for the virial evolution, 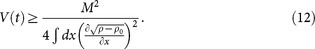
According to Eq. (12), if the virial tends to zero, the gradient norm 
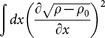
 diverges in order to maintain an invariant mass *M*. This condition leads to the formation of a singularity in the field *ρ* − *ρ*_0_. Equation (10) can be therefore regarded as a sufficient condition for blow-up in the generalized NLS equation (1) with non vanishing boundary conditions. An important observation, stemming from Eq. (12), concerns the role of the background during the collapse. In particular the gradient norm, which drives the collapsing dynamics, does not depend on the background *ρ*_0_. The latter is therefore unaffected by the dynamics and the density evolves by respecting the boundary conditions *ρ*(±∞, *t*) = *ρ*_0_.

## Self-similar analysis and semiclassical argument

The differential inequality (9) can be also employed to calculate the leading order dynamics in both collapsing and non collapsing regimes. We start from the following self-similar form of the field 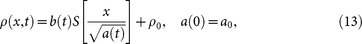
which can be used to extract the scaling dynamics in the unstable regime^3^, and then employ the conservation of the mass *M* to determine the relationship between the peak value *b* and the width (variance) *a* of the field |*ψ*|^2^ − *ρ*_0_, 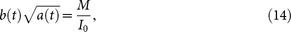
being 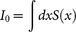. By substituting Eqs. (13) and (14) into Eq. (9), we obtain 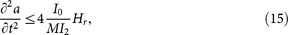
having introduced the constant 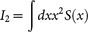 representing the second-order moment of inertia of *S*(*x*) with respect to *x* = 0. By integrating twice Eq. (15) in time, we straightforwardly obtain the following lower bound for the evolution of the peak *b* of |*ψ*|^2^ − *ρ*_0_

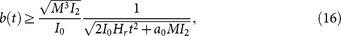
which states that at leading order the field power *b*(*t*) scales as 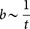 for a non collapsing solution possessing *H_r_* > 0. This scaling has important consequences in the development of shock wave instabilities when the input self-similar parameter is small, i.e., for 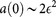 with 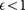. In this case, we can rescale Eq. (1) with 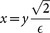 and 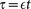, thus obtaining a semiclassical form of the NLS equation with a general nonlinearity: 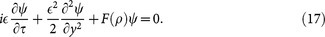
Equation (17) can be casted into a hydrodynamic (or so-called dispersionless) form by applying the Madelung trans-formation 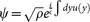 and taking the leading-order terms in 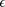, which yields 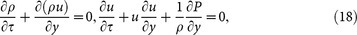
which is physically equivalent to the system (also known as *P*-system) that rules the evolution of a compressible gas flow with density *ρ*, velocity *u* and pressure given through the equation of state *P* = *P*(*ρ*). In our case such equation of state is explicitly given in terms of nonlinearity and density as 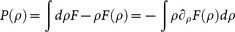, which in turn defines the sound velocity 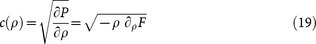
Equivalently Eqs. (18) can be cast in the form of a system of quasi-linear equations *∂_τ_***q** + **A***∂_y_***q** = 0, with **q** ≡ (*ρ*, *u*)*^T^* and 2 × 2 matrix **A** = (*u* *ρ*;−*∂_ρ_F u*), which is diagonalizable in the form 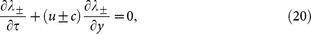
by introducing the Riemann invariants 
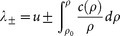
 and the eigenvelocities of the system *v*_±_ = *u* ± *c*, which correspond to the eigenvalues of the matrix **A**. By expanding in Taylor series the nonlinearity *F*(*ρ*) around *ρ*_0_, we obtain 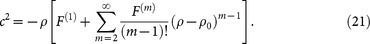
When Eq. (10) is not fulfilled and a collapse is not observed in the dynamics, the field density decreases rapidly (as shown above by the self-similarity argument), and higher-order terms in the sum in Eq. (21) scale as 
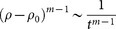
, *m* ≥ 2, thus becoming negligible. As a consequence the velocity *c* is determined by the lowest power of *ρ* in the leading order term 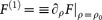. For leading-order cubic nonlinearities where 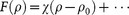, with *χ* = ±1 is the sign of the nonlinearity, we have *F*^(1)^ = *χ* and Eq. (21) yields 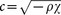. In this case, whenever *χ* = −1 (leading-order cubic nonlinearity of defocusing or repulsive type), the corresponding Riemann velocities 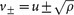 are real and coincides (at leading order), with those pertaining to the integrable (cubic) defocusing NLS system^41^. As a consequence we expect the system to undergo wave-breaking through the development of a gradient catastrophe (formation of vertical front in finite time) from smooth input conditions launched in the system. In the former case the consequent regularization of the dynamics, owing to the dispersive terms neglected in Eqs. (17), occurs in term of a dispersive shock^40^,^41^,^43^,^44^,^45^,^46^,^47^,^48^,^49^,^50^,^51^. Conversely, such argument looses validity in the case *χ* = 1 (focusing cubic nonlinearity), where 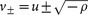 would imply a catastrophe of the elliptic umbilic type (characteristic of systems with dispersionless limit of the elliptic type with complex conjugate eigenvelocities *v*_±_), where *ρ* − *ρ*_0_ increases in time. In such a case higher-order terms in Eq. (21) become important and no conclusive statements on the type of dynamics can be made from such an approach.

In the following section we show, with reference to a specific system, how an arbitrarily small mass perturbation of a solitary waves with non-zero background can lead, depending on its sign, to completely different different decay scenarios implying either collapse or dispersive shock formation.

## Application to cubic-quintic nonlinearity

**Figure 1:**
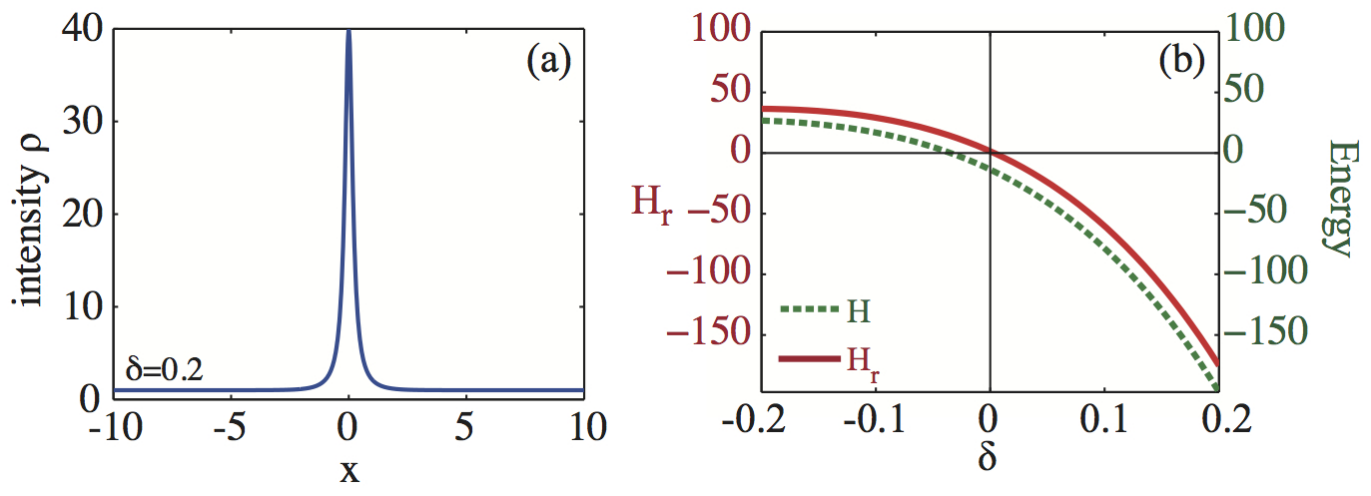
(a) Density distribution *ρ*(*x*, *t* = 0) at fixed perturbation *δ* = 0.2, and (b) Reduced energy functional 
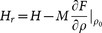
 (solid line) and energy *H* (dashed line) vs. perturbation parameter *δ*, for the perturbed antidark soliton of CQNLS equation, expressed by Eq. (22) with *α* = 0.1 and *ρ*_0_ = 1.

**Figure 2:**
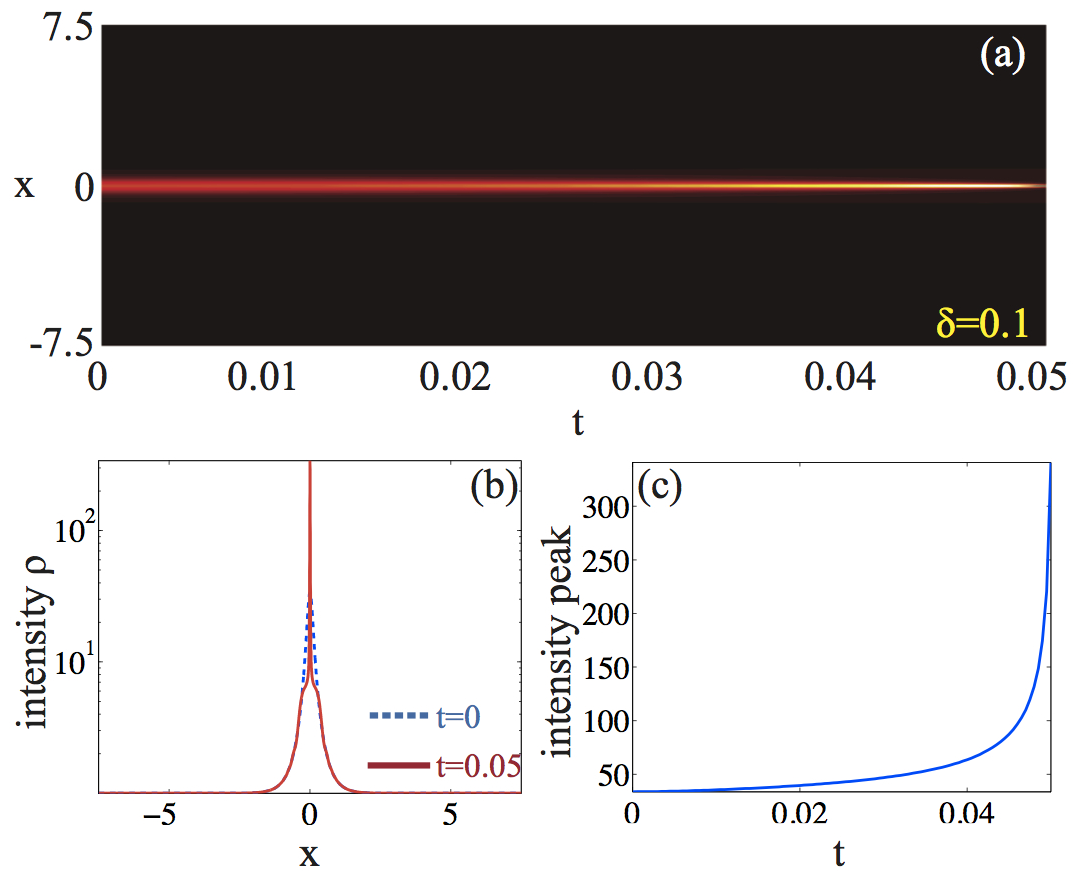
Collapse ruled by the defocusing-focusing CQNLS: (a) color level plot of *ρ* = |*ψ*|^2^ in time-space (*t*, *x*); (b) snapshots of *ρ*(*x*) at *t* = 0 (dashed line) and *t* = 0.049 (solid line); (c) peak intensity (or density) evolution along *t*. Here the quintic coefficient is *α* = 0.1, the background *ρ*_0_ = 1, and we employ a positive-mass perturbation *δ* = 0.1.

In order to illustrate our theoretical findings on a realistic system of physical interest, we consider the CQNLS equation with competing nonlinearities. The CQNLS is a general model for superfluidity^26^, nonlinear optics of saturable nonlinearities or cascading effects^24^,^37^, and systems of quantum condensed gases with elastic two- and three-body interactions^27^,^28^. The regime of interest here is a leading-order (cubic) nonlinearity of the defocusing type saturated by a focusing quintic nonlinearity, which is described by the nonlinear function 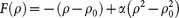, where *α* is a dimensionless parameter describing the strength of the quintic term (responsible for the collapse) over the defocusing cubic nonlinearity. We consider the following input field *ρ*(*x*, *t* = 0): 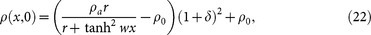
where the small parameter *δ* is introduced to measures a *mass perturbation* of a still (zero velocity) anti-dark soliton *ρ_sol_*(*x*) = *ρ_a_r*/(*r* + tanh^2^ *wx*) [see profile in figure 1(a)], which is an exact solitary solution of the CQNLS provided *r* = *ρ*_0_/(*ρ_a_* − *ρ*_0_), 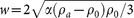, *ρ_a_* = (3 − 4*αρ*_0_)/2*α*^35^. Zero velocity anti-dark solitons of CQNLS *are always unstable* in the spirit of Ref. 38 while their background is stable^35^, thereby being ideal candidate for our theoretical investigation. We begin our analysis by calculating the correspondent reduced energy *H_r_*, shown in figure 1(b). As seen, any positive mass perturbation *δ* > 0 leads to *H_r_* < 0 and, by virtue of Eq. (10), to collapse. In order to numerically ascertain this result, we perform a direct integration of the CQNLS via a fourth order finite difference scheme. In our simulations, we launch Eq. (22) at *t* = 0 and monitor its dynamical evolution in the system for a fixed combination of *α*, *ρ* and a varying *δ*, which defines the mass perturbation of the CQNLS soliton. Our integration scheme is developed within an adaptive fourth order Runge-Kutta method^52^, which automatically adjusts the time resolution in order to maintain good accuracy on the results. The relative error tolerance in the simulation results is 10^−9^. The spatial resolution of the grid was *dx* = 8 · 10^−3^. During each time-step, we also monitored the conserved quantities (2), checking that their value remained practically constant. Figure 2 summarizes our results for instance for *δ* = 0.1, *α* = 0.1 and a background *ρ*_0_ = 1 (similar dynamics is obtained for other choices of values). In perfect agreement with our theory, a positive mass variation leads to a catastrophic collapse of the wave function [see figure 2(a–c)], which is attained at a relatively short distance, as shown in figure 2(c). We also observe, in agreement with Eq. (12), that the background intensity *ρ*_0_ does not change during the collapsing dynamics, remaining stable as also predicted by the linear stability analysis against the growth of periodic modulations^35^.

**Figure 3:**
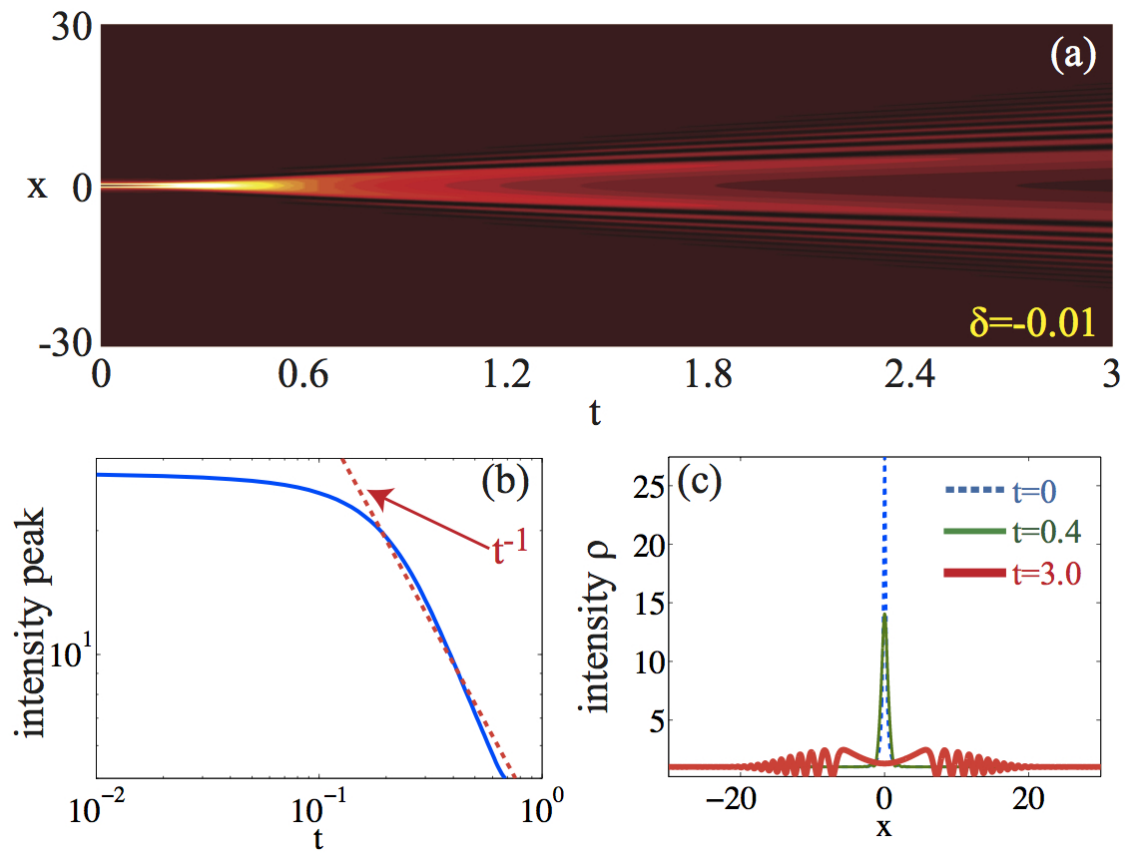
Dispersive shock generation in the defocusing-focusing CQNLS: (a) color level plot of *ρ* = |*ψ*|^2^ in time-space (*x*, *t*); (b) peak intensity (or density) max*_x_*(|*ψ*(*x*, *t*)|^2^) versus *t*; (c) snapshots of *ρ*(*x*) at *t* = 0 (dashed line), *t* = 0.4 (solid thin line) and *t* = 3 (solid thick line). The parameters are as in figure 2 (*α* = 0.1, *ρ*_0_ = 1), except for a negative-mass perturbation *δ* = −0.01.

When the initial soliton mass is conversely decreased, for *δ* < 0, we have *H_r_* > 0 [see figure 1(b)]. In the limit of a perturbative quintic nonlinearity *α* < 1, from Eq. (14) we obtain 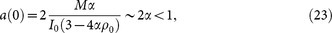
which leads to a semiclassical behavior described by Eqs. (17) – (21) with 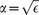. This predicts the generation of dispersive shock waves when *δ* < 0. Figure 3 reports numerical simulations for the case of a negative mass perturbation with *δ* = −0.01 and the same initial parameters (*α* = 0.1, *ρ*_0_ = 1) as in figure 2. In complete agreement with Eq. (16), the intensity amplitude falls off as 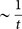 [see figure 3(b)]. This process leads to wave breaking occurring through two symmetric gradient catastrophes (formation of symmetric steep fronts), followed by dispersive shock wave generation, as displayed in figure 3(c). It is worthwhile observing that for *δ* = −0.01 the energy *H* of the field *ψ* is still negative, as indicated by figure 1(b). At variance with the case of bright localized waveform on a zero background, which under the condition *H* < 0, are always collapsing in NLS systems^3^, our analysis demonstrates that a suitable background intensity can arrest the blow-up, leading to a completely different instability scenario where dispersive shocks are generated.

## Conclusions

In conclusion, we investigated wave instability in NLS systems in the presence of a non vanishing background. Through virial identities and semiclassical analysis, we have demonstrated a sufficient criterion for collapse and unveiled a novel instability dynamics characterized by the emission of dispersive shocks. Theoretical findings are verified against numerical simulations on the CQNLS equation, where we unveiled a set of solutions that exhibit a marginal crossover between the two aforementioned regimes. Besides the large interest of the physical community in wave collapse, these results show that a non vanishing background acts an affective mechanism for arresting collapse, leading to a new scenario characterized by the emission of shock waves.

## Author Contributions

A.F. and S.T. conceived the work and developed the theoretical analysis. J.S.T.G. performed the numerical simulations. A.F. and S.T. reviewed and wrote the manuscript.
